# Supplementary material for: New constructions of equality test scheme for cloud-assisted wireless sensor networks
Source: PLoS One. 2021 Oct 25;16(10):e0258746. doi: 10.1371/journal.pone.0258746 (PMC8544876; doi:10.1371/journal.pone.0258746)
Supplement: Attachment — Submitted filename: Response to Reviews.pdf [file pone.0258746.s001.pdf]

Dear Editor,

Thank you very much for your letter and also thank the reviewers for giving us constructive suggestions. Those comments are valuable and very helpful for revising and improving our paper (PONE-D-21-14341). We have studied the comments carefully and have made correction point by point. We hope that these revisions improve the paper such that you now deem it worthy of publication in PLOS ONE. Note that almost all changes are highlighted in yellow in our submitted 'Revised Manuscript with Track Changes' file.

Thank you and best regards.

Yours sincerely,
Huijun Zhu

The following is a point-to-point response to the editor and the two reviewers' comments.

Review Comments to the Author

Reviewer #1:

1. The introduction section should be rewritten in a better way to enhance the readability.

Response:

Thank you for your careful comments. We have rewritten the Introduction thoroughly (please see Page1 and 2).

With the development of Internet of Things (IoT) and the technology of cloud computing [1], cloud-assisted wireless sensor networks (CWSNs) are widely applied in many fields, such as agriculture, military, transportation, medical and other similar fields. Although, CWSNs have extensive applications, but there also exist challenges to be addressed such as reduction of energy consumption. Recently in 2020, Guermazi et al. proposed a method to reduce energy consumption as well as to extend the lifetime of wireless sensor network [2]. For the evaluation models, Cao et al. proposed five intelligent evaluation models and implemented their experiments on the Nearest Closer Protocol with the J-Sim simulation tool [3]. The security of data is another imperative issue. Practically, extensive amount of data is being transmitted and stored on distributed servers, where it may face several threats. Therefore, to protect the confidentiality of such data is particularly important[4]. At present, various cryptographic algorithms are introduced for CWSNs environment. However, private key is necessary to obtain information from the encrypted data, that reduces the availability of data. …… Since, the equality test algorithm is based on a ray. Therefore, comparing the proposal with the schemes based on bilinear pairing, it is simpler and easier to implement.

2. The authors are requested to analyze the security strength of the proposed work with the following papers.

An efficient anonymous authentication and confidentiality preservation schemes for secure communications in wireless body area networks M Azees, P Vijayakumar, M Karuppiah, A Nayyar Wireless Networks 27 (3), 2119-2130.

BBAAS: Blockchain-Based Anonymous Authentication Scheme for Providing Secure Communication in VANETs A Maria, V Pandi, JD Lazarus, M Karuppiah, MS Christo Security and Communication Networks 2021.

Response:

We agree with you. According to your valuable suggestions, we have read some works carefully.

To offer a high level of confidentiality, Maria Azees et al. proposed an efficient affine cipher-based encryption technique [37]. Due to the decentralized nature of blockchain technology, Maria Azees et al. proposed an anonymous authentication scheme based on blockchain [38]. The proposed scheme diminishes the computational cost substantially.

3. The performance analysis part is not sufficient, more results should be added related with equality test.

Thank you sincerely for your comments on our work. According to your valuable suggestions, we have read some works about the schemes with equality test carefully. We have described the performance analysis form encryption and decryption algorithms in references of [23-26] to our works. Details are as follows:

Table 11. The comparison of computational complexity with others

	[23]	[24]	[25]	[26]	Our scheme
C_{Enc}	4E+3I	6E	6E+2P	3E+2I	2E+1I
C_{Dec}	3E+3I	5E	2E+2P+2I	2E+1I	2E+1I

C_{Enc} and C_{Dec}: the computation complexity of algorithms for encryption and decryption; E, P and I: the exponentiation operation, the pairing operation and the inversion operation in the group G.

In Table 11, we present the comparison with the earlier PKEwET schemes while considering the computation complexity of encryption and decryption algorithms. It shows that the presented scheme achieves lower computational complexity.

Again, we sincerely appreciate the time you spent in reviewing our paper.

Reviewer #2: New Constructions of Equality Test Scheme for Cloud-Assisted Wireless Sensor Networks is presented in this paper. It is an interesting topic and contribution is also good. However, I have following queries on this paper:

1. Explain the novelty of your work presented in this work.

Response:

Thank you sincerely for your comments on our work. We have explain the novelty of our work as follows:

In our work, we introduce the idea of public key encryption with equality test into RSA scheme. The proposed scheme fills the gap of RSA algorithm in the context of equality test over ciphertext. To the best of our knowledge, this is the novel algorithm of RSA with equality test. To enhance the security of the scheme, a simple and efficient Fujisaki and Okamoto method is introduced. More precisely, To prove the efficiency of the proposed scheme, the performance analysis is presented on 512, 1024 and 2048 bits. The time of algorithm is compared in context of KenGen , encryption, decryption and test algorithms.

These comparisons validate the claim of scheme efficiency. To simplify the scheme, the equality test algorithm is based on a ray. Therefore, comparing the proposal with the schemes based on bilinear pairing, it is simpler and easier to implement.

2. Paper needs to polish and provide a detailed explanation of theoretical aspects such as conditions and theorems, and practical issues like algorithms, rules and possible applications.

Response:

Thanks for your friendly comments. We have introduced security algorithm of the Table 1 and Table 2 into our work as follows:

As described in Table 1, the KeyGen algorithm takes 1^k and sp as inputs, then, outputs the public key pk and private key sk. A_1 asks for key queries, decryption queries and authorization queries. Then S initiate the challenge phase. S chooses a message m and outputs ciphertext c_t^* by performing encryption algorithm Enc(pk,m). Then, A_1 inquires more queries in phase 2, including key queries, decryption queries and authorization queries. But it must satisfy the requirements of O_4 and O_5. Afterwards, A_1 outputs a message of m, a guessing of c_t^*.

As mentioned in Table 2, the KeyGen algorithm takes 1^k and sp as inputs, then, outputs the public key pk and private key sk. A_2 asks for key queries and decryption queries. A_2 chooses two message m_0 and m_1 for S. Then, S performs challenge phase. S chooses a message m_b ($b \longleftarrow \{0,1\}$) and outputs ciphertext c_t^* by performing encryption algorithm (Enc(pk,m_b)). Then, A_2 inquires more queries in phase 2, such as key queries and decryption queries. But it must satisfy the requirements of O_4 and O_5. Then, A_2 outputs the guess of b.

3. The Introduction section needs to be re-written to improve its quality and readability. Improve the quality of illustrations in this paper and explain those properly.

Following are some of relevant and recent references which need to be discussed in the revised manuscript:

Sensored Semantic Annotation for Traffic Control Based on Knowledge Inference in Video

A Multi-Conditional Proxy Broadcast Re-Encryption Scheme for Sensor Networks

Modeling deep learning neural networks with denotational mathematics in UbiHealth environment

Evaluation models for the nearest closer routing protocol in wireless sensor networks

A novel energy consumption approach to extend the lifetime for wireless sensor network

IoT-based Big Data secure management in the Fog over a 6G Wireless Network

IoT transaction processing through cooperative concurrency control on fog-cloud computing environment

Response:

We agree with you. Again, we sincerely appreciate the time you spent in reviewing our paper. We have rewrited the Introduction and redrawned the picture (see Page 1-3). Moreover we have added some typical studies in Background Knowledge (see Page1 and 4).

With the development of Internet of Things (IoT) and the technology of cloud computing [1], cloud-assisted wireless sensor networks (CWSNs) are widely applied in many fields, such as agriculture, military, transportation, medical and other similar fields. Although, CWSNs have extensive applications, but there also exist challenges to be addressed such as reduction

of energy consumption. Recently in 2020, Guermazi et al. proposed a method to reduce energy consumption as well as to extend the lifetime of wireless sensor network [2]. For the evaluation models, Cao et al. proposed five intelligent evaluation models and implemented their experiments on the Nearest Closer Protocol with the J-Sim simulation tool [3].

In 2020, Li et al. proposed a multi-conditional proxy broadcast re-encryption scheme for sensor networks [41].

4. Many references are with incomplete bibliographic information. This must be corrected.
Thanks for your friendly reminder. We have revised the references carefully, such as:
"Boneh D. CrescenzoG.D, OstrovskyR, PersianoG. Public key encryption with keyword search. In: Cachin C., Camenisch, J.L. (eds.) EUROCRYPT. 2004, 3027, 506-522. " has been changed to " Boneh D. CrescenzoG.D, OstrovskyR, PersianoG. Public key encryption with keyword search. In: Cachin C., Camenisch, J.L. (eds.) EUROCRYPT. 2004, 3027, 506-522. ".

5. It seems that the contribution points of the article are a little bit few. After or in the section of Motivation, it is recommended that the authors summarize the contribution points of their work, which clearly demonstrate the innovations.
Thank you sincerely for your comments on our work. We have rewrited the contribution of our work as follows:

The idea of public key encryption with equality test is introduced into RSA scheme. The proposed scheme fills the gap of RSA algorithm in the context of equality test over ciphertext. The major target of this paper is to make the RSA algorithm enjoying the equality test of ciphertexts. To the best of our knowledge, this is the novel algorithm of RSA with equality test.

A simple and efficient Fujisaki and Okamoto method is introduced to enhance the security of the proposed scheme. More precisely, a semantically secure public-key encryption scheme against passive adversaries is improved to a non-malleable public-key encryption scheme against adaptive chosen ciphertext attacks in the random oracle model.

To prove the efficiency of the proposed scheme, the performance analysis is presented on 512, 1024 and 2048 bits. The time of algorithm is compared in context of KenGen , encryption, decryption and test algorithms. These comparisons validate the claim of scheme efficiency.

Since, the equality test algorithm is based on a ray. Therefore, comparing the proposal with the schemes based on bilinear pairing, it is simpler and easier to implement.

6. There are many English and grammatical issues in the paper which need to be rectified.
We agree with you. We have checked the manuscript carefully again both in English and in depth to improve the quality of our manuscript. Here we list some corrections (for an incomplete list) as follows:
(1) " Although the application of CWSNs is very extensive, it still faces many challenges, especially the energy consumed. " has been changed to " Although, CWSNs have extensive applications, but there also exist challenges to be addressed such as reduction of energy consupmtion. "

(2) “ Practically, abundant of data are transmitting that is stored on distributed servers. ” has been changed to “ extensive amount of data is being transmitted and stored on distributed servers. ”
(3) “. These sensitive data are faced with various threats.” has been changed to “, where it may face several threats.”
(4) “ When the PKEwET scheme is applied in CWSNs, such as in Fig.1, ” has been changed to “The application scenario of PKEwET in context of CWSNs is depicted in Fig. 1.”
(5) “ To enhance the security of the scheme, the method of Fujisaki and Okamoto is introduced into the proposed scheme. ” has been changed to “For security enhancement, Fujisaki and Okamoto is introduced into the proposed scheme.”
(6) “ chosenciphertext ” has been changed to “ chosen ciphertext ”.
(7) “We can see that the proposed scheme is very efficient.” has been changed to “ These comparisons validate the claim of scheme afficiency. ”
(8) “ The time of algorithm is described from KenGen algorithm, encryption algorithm, decryption algorithm and test algorithm. ” has been changed to “ The time of algorithm is compared in context of KenGen, encryption, decryption and test algorithms. ”
Again, we sincerely appreciate the time you spent in reviewing our paper.
